# Improving the effectiveness and efficiency of outpatient services: a scoping review of interventions at the primary–secondary care interface

**DOI:** 10.1177/1355819616648982

**Published:** 2016-05-10

**Authors:** Eleanor M Winpenny, Céline Miani, Emma Pitchforth, Sarah King, Martin Roland

**Affiliations:** 1 Cambridge Centre for Health Services Research, RAND Europe, Cambridge, UK; 2 RAND Professor of Health Services Research, Cambridge Centre for Health Services Research, University of Cambridge, UK

**Keywords:** efficiency, outpatient, primary care, referral

## Abstract

**Objectives:**

Variation in patterns of referral from primary care can lead to inappropriate overuse or underuse of specialist resources. Our aim was to review the literature on strategies involving primary care that are designed to improve the effectiveness and efficiency of outpatient services.

**Methods:**

A scoping review to update a review published in 2006. We conducted a systematic literature search and qualitative evidence synthesis of studies across five intervention domains: transfer of services from hospital to primary care; relocation of hospital services to primary care; joint working between primary care practitioners and specialists; interventions to change the referral behaviour of primary care practitioners and interventions to change patient behaviour.

**Results:**

The 183 studies published since 2005, taken with the findings of the previous review, suggest that transfer of services from secondary to primary care and strategies aimed at changing referral behaviour of primary care clinicians can be effective in reducing outpatient referrals and in increasing the appropriateness of referrals. Availability of specialist advice to primary care practitioners by email or phone and use of store-and-forward telemedicine also show potential for reducing outpatient referrals and hence reducing costs. There was little evidence of a beneficial effect of relocation of specialists to primary care, or joint primary/secondary care management of patients on outpatient referrals. Across all intervention categories there was little evidence available on cost-effectiveness.

**Conclusions:**

There are a number of promising interventions which may improve the effectiveness and efficiency of outpatient services, including making it easier for primary care clinicians and specialists to discuss patients by email or phone. There remain substantial gaps in the evidence, particularly on cost-effectiveness, and new interventions should continue to be evaluated as they are implemented more widely. A move for specialists to work in the community is unlikely to be cost-effective without enhancing primary care clinicians’ skills through education or joint consultations with complex patients.

## Introduction

One important role of general practitioners (GPs) in the UK National Health Service (NHS) is to decide when patients need to be referred to specialists. Over-referral is costly and may expose patients to unnecessary harm, while under-referral deprives patients of treatments from which they could benefit. There is known to be wide and unexplained variation in the referral rates of GPs which can lead to overuse or underuse of specialist resources.^1^ There have been many attempts to improve the effectiveness and efficiency of this process, summarized in a review by Roland et al.^2,3^ It found that transferring services from secondary to primary care and strategies intended to change the referral behaviour of GPs were often effective in improving outpatient effectiveness and efficiency. However, relocating specialists to primary care and developing joint working between primary and secondary clinicians were largely ineffective.

In recent years, the need to improve efficiency of health services has increased. One initiative in England in 2012 was the transfer of responsibility to clinical commissioning groups (CCGs). This required GPs working as part of CCGs to balance their responsibility to their patients with a responsibility to manage NHS budgets. More recently, a number of new models of care, intended to provide more integrated care as well as increasing efficiency have been encouraged.^4^ Similar initiatives are ongoing in other countries.^5^

Our aim was to update the earlier review on primary care strategies to improve the effectiveness and efficiency of outpatient services. While our primary interest was to identify evidence for England and Wales, we also drew upon international evidence so our findings would be relevant for other countries. We considered the four domains of interventions considered in the previous review, plus an additional one:

- Transfer: The substitution of services delivered by specialists for services delivered by primary care clinicians.
- Relocation: Shifting the venue of specialist care from outpatient clinics to primary care without changing the people who deliver the service.
- Liaison: Joint working between specialists and primary care clinicians to provide care to individual patients.
- Professional behaviour change: Interventions intended to change the referral behaviour of primary care clinicians, including referral guidelines, audit and feedback, professional education and financial incentives.
- Patient behaviour change: Decision aids and aids to patient choice designed to influence decisions about referral to and discharge from specialist clinics.

## Methods

We conducted a scoping review to map rapidly the key concepts underpinning a research area and the main sources and types of evidence available.^6^ A more detailed report of our methods and findings has been published elsewhere.^7^ We considered interventions to improve outpatient efficiency in each of the five domains.

### Data sources and search strategy

MEDLINE, EMBASE, HMIC Health Management and Policy database and the King’s Fund database of grey literature were searched for literature published between February 2005 and April 2014 using terms relating to primary care, interventions considered within the five intervention domains and outcomes such as outpatient referral or appointment.

### Study selection

To be eligible for inclusion, studies had to evaluate interventions in primary care that had the potential to improve effectiveness and efficiency of outpatient services. Outcomes of interest included, but were not limited to, patient access (including waiting times), referral rates, patient outcomes, service outcomes, physician outcomes and costs. Studies had to be conducted in a high-income country and had to report on an intervention that was potentially transferable to the NHS. As defined by Arksey and O’Malley^6^, a scoping review synthesizes evidence across a broad topic area where many different study designs may be applicable, and as such ‘quality assessment does not form part of the scoping review remit’. Therefore, all types of observational and experimental studies were included, as well as reviews. We did not formally assess the quality of studies, and no studies were excluded based on study quality. The types of study which form the basis for our conclusion in each category provide an indication of the extent and strength of evidence (Table 1).

**Table 1.** Key findings on effectiveness and cost-effectiveness across the intervention categories.

Model	Type of working arrangement	Key findings on intervention effectiveness	Key findings on cost- effectiveness	Study type
Transfer: The substitution of services delivered by hospital clinicians for services delivered by primary care clinicians	Minor surgery in primary care	Minor surgery carried out in general practice can be safe and effective, but this depends critically on the skill and training of the operator.	The cost-effectiveness of minor surgery carried out in general practice is likely to depend on local payment/contractual arrangements.	Six studies: one RCT, four non-controlled observational studies, one economic evaluation
	Medical care of chronic disease in primary care	With adequate supervision and training, a wide range of conditions can be managed in primary care both safely and effectively.	Few studies examine the cost-effectiveness of transferring chronic disease management from secondary to primary care.	16 studies: 3 RCTs, 4 before-and-after studies, 6 non-controlled observational studies, 1 review (non-systematic), 2 modelling studies
	Intermediate care (e.g. GPs with special interests)	GPSIs can provide an effective addition to specialist outpatient services, associated with high levels of patient satisfaction.	Whether GPSIs provide a cost-effective alternative to outpatient clinics remains unclear and may depend on local service configuration and contractual arrangements. The provision of GPSI services may change GP’s referral thresholds and result in ‘supply induced demand’.	10 studies: 2 RCTs, 2 before-and-after studies, 2 non-randomized controlled studies, 1 economic evaluation, 2 non-controlled observational studies, 1 discussion paper
	Outpatient discharge to primary care	GPs can follow up patients across a range of diagnostic groups as an alternative to hospital follow-up It is important to ensure that general practices have the administrative support and specialist support needed.	We found limited empirical evidence on cost-effectiveness, although the evidence available suggested some reduction in costs. A modelling study suggested considerable cost savings could be made from transfer of cancer follow-up to primary care.	13 studies: 1 systematic review, 4 RCTs, 7 non-controlled observational studies, 1 modelling study
	Direct access by GPs to diagnostic tests and investigations	Patients value being able to have tests ordered directly by their GP, especially where tests are locally available Especially for complex tests such as MRI and CT, increased convenience to patients may need to be balanced against the greater efficiency of tests being carried out in a centralized location.	The costs of providing services in the community compared to in hospital are not commonly reported.	25 studies: 2 RCTs, 4 non-randomized controlled studies, 3 before-and-after studies, 14 non-controlled observational studies, 2 economic evaluations
	Direct access by GPs to specialist services	In some cases, the benefits of bypassing an unnecessary specialist referral are clear-cut. Direct access to some services (e.g. physical therapies for musculoskeletal problems) produces a substantial increase in demand.	Cost-effectiveness of direct access to services as an alternative to referral is not clear.	Seven studies: one RCT, one non-randomized controlled study, three before-and-after studies, two non-controlled observational study
Relocation: Shifting the venue of specialist care from outpatient clinics to primary care without changing the people who deliver the service	Shifted outpatient clinic	Community clinics are popular with patients, may reduce waiting times and are reported to reduce attendances at the hospital outpatient clinic. Community clinics may provide educational opportunities for primary care staff; however, we did not find conclusive evidence to update conclusions from the previous review that this generally did not occur.	There is limited evidence on effect on costs. Community clinics may increase costs due to little difference in costs between community and hospital clinics and the potential for increased total referrals.	Four studies: one non-randomized controlled study, one before-and-after study, one non-controlled observational study, one discussion paper
	Attachment of specialists to primary care teams	Specialist attachments to primary care teams have a stronger educational focus than shifted outpatient clinics. Few formal evaluations of this type of attachment have been reported: they appear costly and often have depended on the enthusiasm of individual specialists to undertake this type of work.	Very limited evidence: one study suggests an overall saving to the healthy economy.	Two studies: two non-controlled observational studies
	Community mental health teams	Collaborative models of mental health care are likely to be effective across a wide spectrum of disorders. Community mental health teams are likely to be most effective when there are regular opportunities for face to face contact between mental health workers and the primary care practice team.	There is little evidence on the cost-effectiveness of different models of care and, especially given the diversity of local arrangements, little to guide local commissioners on the optimum configuration of services.	10 studies: 4 systematic reviews, 1 review (non-systematic), 2 RCTs, 3 non-controlled observational studies
				
	Telemedicine	While in countries with very remote rural areas, video-consultation may be viable, it is unlikely that video-consultations will be a cost-effective alternative to outpatient clinics in England. Store-and-forward services for images of skin conditions show promise, although may be of less value in suspected skin cancer.	Very few evaluations of telemedicine present robust economic analyses.	32 studies: 1 systematic review, 2 reviews (non-systematic), 3 RCTs, 4 non-randomized controlled studies, 2 before-and-after studies, 17 non-controlled observational studies, 3 economic evaluations
Liaison: Joint working between specialists and primary care practitioners to provide care to individual patients	Shared care, including consultation liaison	Care can be given in primary care using shared care protocols across a wide range of conditions without loss of quality. Shared care may not improve care or reduce duplication where there is lack of agreement as to who will be doing what.	Compared to outpatient visits, cost savings to patients can be considerable, but savings to the health service are less clear-cut. Some studies show net savings by moving care from outpatient clinics to a shared care model, but such savings are not universal.	Eight studies: two systematic reviews, two non-randomized controlled studies, three non-controlled observational studies, one economic evaluation
Professional behaviour change: Interventions intended to change the referral behaviour of primary care practitioners	Guidelines	Guidelines, audit and feedback and professional education programmes are all relatively ineffective on their own but may be effective when combined, or linked with other interventions. Guidelines are increasingly incorporated into referral proformas which have to be completed as part of the referral process. Guidelines may increase or reduce numbers of referrals: interventions aimed at changing professional behaviour should be aimed at increasing the appropriateness of referrals rather than at demand management.	No evidence on cost-effectiveness.	20 studies: 2 systematic reviews, 3 RCTs, 3 non-randomized controlled studies, 8 before-and-after studies, 4 non-controlled observational studies
	Audit and feedback	Despite the widespread use by primary care trusts and clinical commissioning groups of feedback to GPs on their referral data, we did not find sufficient additional evidence to draw conclusions about the value of such feedback.	No evidence on cost-effectiveness.	One before-and-after study
	Professional education, including academic detailing	Professional education may be effective in increasing appropriateness of referral but may depend upon the degree of intensiveness of the intervention, which varied from intensive specialist support to single educational events. There is a clear tension between education at an intensity which may not be sustainable, and more modest interventions that appear less effective.	No evidence on cost-effectiveness.	18 studies: 1 systematic review, 5 RCTs, 10 before-and-after studies, 2 non-controlled observational studies
	In-house review of referrals	Evidence on in-house referral schemes is very limited. The weak evidence that exists supports this approach in terms of reducing referrals and suggests that the approach is acceptable to patients.	No evidence on cost-effectiveness.	Four studies: one non-randomized controlled study, two before-and-after studies, one non-controlled observational study
	Referral management centres	There is very limited evidence published on the effectiveness of referral management centres.	The evidence that exists is equivocal and suggests that reduction in referrals by referral management centres is less likely to represent value for money than the use of more passive alternatives such as in-house review of referrals.	Six studies: one review, one non-randomized controlled study, one before-and-after-study, three non-controlled observational studies
				
	Financial incentives	A number of financial incentives have been introduced through the QOF which indirectly increased the number of referrals made by GPs for specific conditions. No studies were found that reported on the direct incentive introduced through the QOF to review outpatient referrals.	No evidence on cost-effectiveness.	Six studies: one systematic review, four before-and-after studies, one non-controlled observational study
	Requests for specialist advice by email or phone	Studies where GPs can obtain specialist advice by phone or email suggest there is substantial opportunity to reduce the number of patients who are seen in outpatient clinics.	Only two studies report on costs, but both report an overall cost saving from the intervention, as a result of reductions in secondary care costs.	Eight studies: two before-and-after studies, six non-controlled observational studies
Patient behaviour change	Decision aids and aids to patient choice designed to influence decisions about referral to and discharge from specialist clinics	Insufficient evidence was found to draw conclusions.	No evidence on cost-effectiveness.	One RCT

GPs: general practitioners; GPSIs: GPs with a special interest; RCTs: randomized controlled trials; MRI: magnetic resonance imaging; CT: computed tomography; QOF: Quality and Outcomes Framework.

Titles and abstracts of studies identified by the searches were assessed for inclusion by two researchers. Those selected were reassessed by a third researcher. Full texts were then assessed against the inclusion and exclusion criteria by four researchers. Disagreements or uncertainties were resolved by discussion within the research team.

### Data extraction and analysis

Study data were extracted by four reviewers into a template. Findings for each intervention category were then summarized by one reviewer, and the summaries discussed and modified by the research team as necessary, to generate an overall conclusion about the impact on outpatient effectiveness and efficiency.

## Results

Our search identified 21,135 records, from which 183 were eligible for inclusion. Only a few were controlled trials or systematic literature reviews, with much of the literature comprising observational studies (Figure S1, available online). We provide an overview of the principal findings for each intervention category with examples of the studies which form the basis of each of our conclusions. Where a systematic review or randomised controlled trial (RCT) is available, we include at least one of these as the example; otherwise we have selected references which we believe provide the strongest support (Table 1). A number of studies report interventions within more than one category.

**Figure 1.**
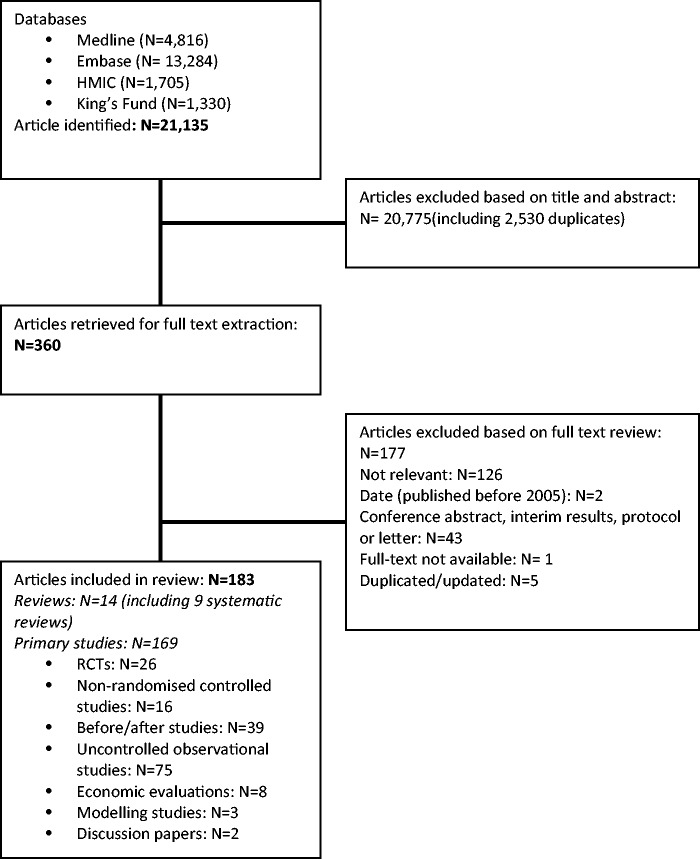
PRISMA flow diagram. RCTs: ▪.

### Transferring care to primary care

Our review covered six interventions in which services or elements of services were transferred from secondary to primary care:

- Minor surgery (six studies)
- Medical care of chronic disease (16 studies)
- GPs with a special interest (10 studies)
- Outpatient discharge to primary care (13 studies)
- Direct access by GPs to diagnostic tests and investigations (25 studies)
- Direct access by GPs to specialist services (seven studies)

The evidence suggests that both minor surgery and care for a range of chronic diseases (including diabetes, asthma, alcohol dependence and chronic obstructive pulmonary disease) can be provided safely and effectively in primary care, as well as some evidence that additional primary care resources can reduce secondary care utilization.^8–10^ There is also evidence that GPs can follow up patients across a range of diagnostic groups, including cancer, dementia and renal failure, as an alternative to hospital follow-up.

For surgery, medical care and postacute follow-up, it is important that primary care practitioners have additional training and support where needed. The previous review^3^ suggested that quality of surgery in general practice was low compared to outpatients, and we found one further study that reinforced that view.^14^ However, several other studies that evaluated more specialist, highly-trained GPs (GPs with a special interest (GPSIs)) found no difference from hospital doctors.^11,12^ Support may be required to ensure that GPs and nurses are confident with their additional roles,^13^ and that support from specialists is available when queries or problems arise.^14^ Administrative support, such as electronic decision support tools may be needed to ensure that follow-up protocols can be followed reliably.^15^

A large number of studies have been conducted that examine the effects of giving GPs access to a wider range of diagnostic tests including magnetic resonance imaging, computed tomography, diagnosis and management of deep vein thrombosis, retinal photography for diabetic retinopathy and gynaecological ultrasound. Especially when combined with a referral protocol, it is evident that GPs can make effective use of a wide range of diagnostic facilities. However, several studies of imaging showed a very low rate of positive findings,^10,16^ suggesting that investigations may be undertaken when not needed, although in some cases these investigations may still allow avoidance of outpatient referrals.

Seven papers reported on direct access of GPs to specialist services, bypassing the need to see a consultant. Some showed benefits such as improved access and improved patient satisfaction from direct referral to audiology.^17^ In one example of direct access to a low visual aid clinic, direct access resulted in an increase in referrals, possibly indicating that unmet need was being addressed.^18^ While the costs of transferring services are not frequently reported, cost-effectiveness is likely to depend upon local contractual arrangements, and differ by condition and procedure.

### Relocation of specialist services to primary care settings

Our review covered four interventions in which services or elements of services were relocated from a secondary care to primary care setting, with the provider remaining a specialist. These were:

- Shifted outpatient clinics (four studies)
- Specialist attachment to primary care teams (two studies)
- Community mental health teams (10 studies)
- Telemedicine (32 studies)

Although studies of shifted outpatient clinics reported that patient satisfaction was high and waiting times were reduced in some cases, the effects on numbers of referrals to both community and hospital services and on costs were unclear.^19,20^ One new study on attachment of a specialist doctor or nurse to primary care teams reported a reduction in referrals and cost for a clinic run by a GP with special interest together with a specialist allergy nurse compared with referral to a consultant outpatient clinic.^21^ A second study described substantial educational benefits for GPs where a consultant visited practices and conducted joint consultations with patients, although this study did not address economic aspects of the intervention.^22^

Collaborative models of mental health care appear effective across a wide spectrum of conditions. The evidence suggests that these may be most effective when there are regular opportunities for face-to-face contact between members of the mental health team and the primary care team.^23^ There is some evidence that collaborative mental health care can lead to a reduction in further specialist referral^24^ but little evidence on the cost-effectiveness of different models of care.^25^

Telemedicine ‘store-and-forward’ services have been developed in cardiology, dermatology, ophthalmology and oncology allowing for digital images or other test results be taken locally and sent to specialists for feedback. While services are not physically relocated, this technology allows specialists to provide services within primary care. Teledermatology for images of skin conditions have been shown in most cases to give accurate diagnoses and show potential to reduce referrals,^26^ although they appear to be of less value in cases of suspected skin cancer.^27^ Few evaluations of telemedicine present robust economic analyses.

### Joint management of patients by primary and secondary care clinicians

We found eight new studies relating to shared care of patients, including two systematic reviews.^28,29^ Overall, these studies suggest that care can be provided in primary care using shared care protocols without loss of quality. Cost savings to patients can be considerable (e.g. transport costs), but savings to the health service are less clear. There are studies which show net savings by moving from outpatient clinics to a shared care model, but such savings are not universal and may depend on the nature of the shared care arrangement.^28,30^

### Professional behaviour change to reduce rates of referral from primary to secondary care

Our review covered seven interventions which attempted to change professional referral behaviour:

- Guidelines, including referral proformas (20 studies)
- Audit and feedback (one study)
- Professional education including academic detailing (18 studies)
- In-house review of referrals (four studies)
- Referral management centres (six studies)
- Financial incentives (six studies)
- Advice requests (eight studies)

Several studies (including one systematic review) that considered passive dissemination of referral guidelines, showed them to be ineffective.^31,32^ However, other studies suggested that guidelines can be effective when used in combination with other interventions such as structured referral proformas.^33,34^ Studies suggested that both guidelines and professional education have potential to increase as well as decrease numbers of referrals,^35–37^ and therefore interventions should be aimed at increasing the appropriateness of referrals rather than at demand management. Many studies found that educational programmes were associated with an increase in the appropriateness of referrals,^38–40^ although some found no impact.^41^

From a limited evidence base, feedback on referrals, either internal from within the GP practice (in-house review of referrals)^42^ or external^43^ can improve the appropriateness of referrals. Evidence for the impact of referral management centres on reducing outpatient referrals was weak. Two studies concluded they were less likely to represent value for money than other approaches such as in-house review of referrals.^44,45^

Studies on financial incentives focussed on either direct incentives to reduce referrals or on the secondary effects of other incentives. Findings from a review of interventions targeting outpatient referrals suggested that GP fundholding, which created a financial incentive for GPs to reduce referrals for conditions for which they held the budget, resulted in a small reduction in referrals for these conditions.^33^ The Quality and Outcomes Framework (QOF), which provided incentives for chronic disease management, appeared to increase referrals in response to particular incentives, for example referrals to a diabetic clinic increased after tighter targets for blood sugar control were introduced into the QOF.^46^

A number of studies have evaluated interventions which enable GPs to get email or phone advice from specialists without the need for patients to attend a face-to-face consultation with the specialist. These studies, which cover areas such as endocrinology and neurology, suggest that there is substantial opportunity to reduce the number of patients who are seen in outpatient clinics. Email or phone advice allowed GPs to avoid referral to outpatient consultation^47,48^ and reduce costs. One study reported that 88% of virtual consultations were resolved without requiring a hospital visit, alongside a reduction of inappropriate referrals from 25% to 10% after introduction of the virtual consultation system.^47^

### Patient behaviour change

Only one new study, an RCT, assessed the effect of patient coaching or patient decision aids.^49^ Patients were offered coaching through an online portal regarding how to discuss their condition with their primary care doctor. Patients in the coaching group were more likely to be referred to a specialist than those who had not received coaching. No difference, however, was found in diagnosis, management or outcomes between groups, suggesting that this increase in referrals may not have been helpful for patient treatment.

## Discussion

We identified 183 new studies and reviews published since 2005, across five intervention domains. We kept the same conceptual framework for describing interventions at the primary–secondary interface as the previous review, namely transferring care to primary care, relocation of specialist services to primary care settings, joint management by primary and secondary care clinicians and interventions designed to change professional behaviour, adding an additional domain of interventions for patient behaviour change. Behind many of the studies are assumptions about the benefits of proposed changes such as that overall demand will not change when services are made more available or that services in the community are cheaper. There is a lack of evidence for some of these assumptions with little measurement of the impact of interventions on the health care system and a lack of evaluation of cost-effectiveness. These need to be addressed in future research.

Our findings are consistent with those from the previous review, suggesting that transfer of services from secondary to primary care, and strategies aimed at changing the referral behaviour of primary care clinicians, can be effective in either reducing outpatient referrals or increasing the appropriateness of referrals made. New studies on increasing the availability of specialist advice to primary care clinicians by email or phone, and, to some extent, use of store-and-forward telemedicine, showed potential for reducing outpatient referrals and hence reducing costs. Across all the intervention domains evaluated, there was very little evidence available on cost-effectiveness. Further research is needed to clarify whether even those interventions where there is good evidence for a reduction in outpatient referrals are cost-effective, when costs across the whole health system are taken into account.

There was new evidence on the effect of relocating specialists to primary care or joint primary/secondary care management of patients, suggesting that there can be educational advantages for GPs from these interventions. The previous review concluded that relocation of specialists into primary care, although associated with improved access, was not effective in reducing outpatient attendances. Indeed, studies suggest that, by making services more accessible, there is potential to increase demand by reducing referral thresholds, and there may be a loss of efficiency when specialists do clinics in dispersed community settings. There is evidence of reduced referrals when the specialist (doctor or nurse) carries out joint clinics with a GP with a special interest or provides educational input in some other way. In some new models of care proposed in England,^6^ it is therefore important that specialists do more than simply see patients in community settings; new arrangements need to add value through increased interaction with clinicians working in primary care if they are to prove cost-effective.

As a scoping review, this study does not include a formal quality assessment of studies, and as such does not allow us to provide a definitive analysis of the strength of evidence available to support each intervention. However, it did allow us to include a wide range of study designs, albeit with recommendations that can only be cautious when the evidence base is weak. This methodology also allows us to describe the breadth of interventions which have been investigated and provide some assessment of their effectiveness from the diverse evidence available. Further systematic assessments of specific interventions may be required where these do not already exist.

This type of review also allows gaps in the research literature to be identified. Studies are needed which employ rigorous experimental designs, supported by qualitative research to assess factors which may have an impact on implementation in other settings. One particular area needing research is assessment of the cost-effectiveness of interventions, taking into account costs to the whole health system. Cost-effectiveness will depend upon local reimbursement arrangements, or tariffs set up for new initiatives such as ‘advice referral’. This information will be important to those designing new service models and commissioning services.

Two motives drive interventions to change care at the primary–secondary interface: to provide care closer to a patient’s home,^50^ thus increasing convenience and to improve overall cost-effectiveness. The literature shows that high-quality care in the community can be provided for many conditions and is popular with patients. However, there are only a few robust economic evaluations, especially those which look at the whole health care system, and some studies suggest that community care may in some circumstances be more expensive due to loss of economies of scale, increased cost of staff and the possibility that providing more convenient access to care may increase demand. Outcomes and costs will also depend on whether or not specialist staff change the type of work they do when they work in the community, for example through closer interaction with primary care staff. A further issue is the distinction between price and cost: economic evaluations tend to use standard published NHS costs, but these may in some circumstances be undercut by the prices charged by NHS providers. This may explain discrepancies where purchasers claim cost savings in situations where the literature finds little evidence of cost-effectiveness. The need for better economic evaluations is one of the key conclusions from this review.

## Supplementary Material

Supplementary material

## Supplementary Material

Figure S1 is available online at http://hsr.sagepub.com/supplemental
