# Supplementary figures and images for: Improving the effectiveness and efficiency of outpatient services: a scoping review of interventions at the primary–secondary care interface
Source: J Health Serv Res Policy. 2016 May 10;22(1):53–64. doi: 10.1177/1355819616648982 (PMC5482389; doi:10.1177/1355819616648982)

Figure S1: PRISMA flow diagram

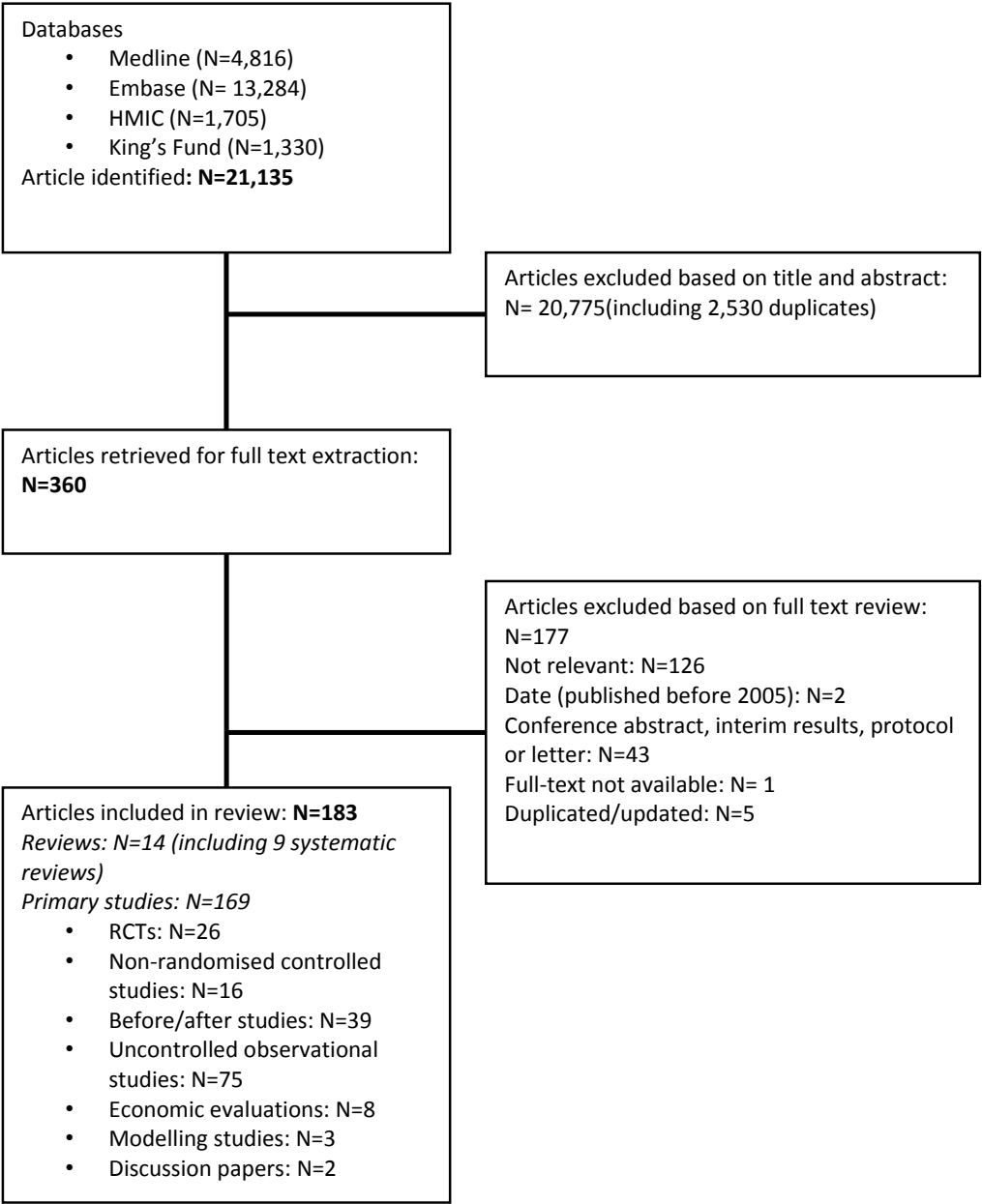

RCTs: Randomised controlled trial.

Supplement: Supplementary material [file HSR648982_supplementary_material.pdf]
